# A contribution to *Porogramme* (*Polyporaceae*, *Agaricomycetes*) and related genera

**DOI:** 10.1186/s43008-023-00110-z

**Published:** 2023-03-07

**Authors:** Wei-Lin Mao, Ying-Da Wu, Hong-Gao Liu, Yuan Yuan, Yu-Cheng Dai

**Affiliations:** 1 School of Ecology and Nature Conservation, Beijing Forestry University, Beijing, 100083 China; 2 Key Laboratory of Forest and Grassland Fire Risk Prevention, Ministry of Emergency Management, China Fire and Rescue Institute, Beijing, 102202 China; 3 Zhaotong University, Zhaotong, 657000 China

**Keywords:** *Polyporaceae*, Phylogeny, Taxonomy, Wood-rotting fungi, Five new taxa

## Abstract

The polypores with shallow pores from tropical Asia and America are studied. Our molecular phylogeny based on the internal transcribed spacer (ITS), the large subunit nuclear ribosomal RNA gene (nLSU), the translation elongation factor 1-α gene (TEF1), and the largest subunit of RNA polymerase II (RPB1) demonstrates six clades are formed among *Porogramme* and related genera. Two new genera, *Cyanoporus* and *Pseudogrammothele*, are established, and the six clades represent *Porogramme, Cyanoporus, Grammothele, Epithele, Theleporus*, and *Pseudogrammothele*, respectively. The molecular clock analyses estimate the divergence times of the six clades based on a dataset (ITS + LSU + TEF1 + RPB1 + RPB2), and we recognize the mean stem ages of the six genera are earlier than 50 Mya. Three new species in *Porogramme* were morphologically and phylogenetically confirmed, and they are described as *P. austroasiana*, *P. cylindrica*, and *P. yunnanensis*. Phylogenetic analysis shows that type species of *Tinctoporellus* and *Porogramme* are nested in the same clade, and *Tinctoporellus* is treated as a synonym of *Porogramme.* Based on our phylogeny, twelve new combinations are proposed, and the differences between the new species and similar or related species are discussed.

## Introduction

*Porogramme*, typified by *P. albocincta*, is characterized by the resupinate, bluish gray, reddish to almost black basidiome with an irpicoid to poroid hymenophore, hymenium restricted to the base of tubes, a monomitic hyphal system, generative hyphae with clamp connections and dextrinoid, the absence of cystidia and dendrohyphidia, ellipsoid to cylindrical, thin-walled, neither amyloid nor dextrinoid basidiospores, a white rot ecology, with the substrate becoming reddish beneath the basidiomes (Ryvarden and Johansen 1980).

*Theleporus* (typified by *T. cretaceus*), *Grammothele* (typified by *G. lineata*), *Epithele* (typified by *E. typhae*), and *Porogramme* are similar in sharing hymenia restricted to the base of tubes, and these genera are traditionally called corticioid fungi (Ryvarden 1979; Ryvarden and Johansen 1980; Larsson 2007). *Tinctoporellus*, typified by *T. epimiltinus*, is similar to above four genera, but differs by the hymenium being present at both the base and vertical wall of tubes, and was considered a true polypore (Ryvarden 1979). *Epithele* differs from *Grammothele*, *Theleporus*, and *Porogramme* by the smooth hymenophore and thick-walled basidiospores, *Theleporus* differs from *Grammothele* and *Porogramme* in the pale basidiome and in not reddening substrate. *Grammothele* differs from *Porogramme* by the presence of dendrohyphidia.

Species in *Porogramme*, *Theleporus*, *Grammothele*, *Epithele*, and *Tinctoporellus* are mostly distributed in tropical or subtropical areas (Ryvarden and Johansen 1980; Wu et al. 2022). Recently, more new species in above genera were described (Yuan and Wan 2012; Zhou and Dai 2012; Nakasone 2013; Ryvarden 2015, 2018, 2019; Yuan 2015; Wu et al. 2016; Hyde et al. 2019; Decock and Ryvarden 2020, 2021). The five genera were previously proved to belong to *Polyporaceae* based on molecular phylogeny (Binder et al. 2005; Zhou and Dai 2012; Justo et al. 2017). However, phylogenetic relationships between *Porogramme* and *Tinctoporellus*, *Epithele, Grammothele* and *Theleporus* were not analyzed, and their species diversity is not well known.

Based on samples from Brazil, China, Malaysia, Singapore, Sri Lanka, and Vietnam, phylogenetic analyses on *Porogramme* and *Tinctoporellus*, *Epithele, Grammothele* and *Theleporus* are carried out. Three new species belonging to *Porogramme* are detected, and their illustrated description are given, the new definition of the genus is outlined. In addition, two new genera and twelve new combinations are proposed.

## Materials and methods

### Morphological studies

Voucher specimens examined are deposited in the collection of the Institute of Microbiology, Beijing Forestry University (BJFC). Morphological descriptions are based on field notes and voucher specimens. Sections of basidiome were studied microscopically according to Cui et al. (2019), and examined at 1000 × using a Nikon Eclipse 80i microscope.

The following abbreviations are used in the descriptions: KOH = 5% potassium hydroxide, IKI = Melzer’s reagent, IKI– = neither amyloid nor dextrinoid, CB = Cotton Blue, CB +  = cyanophilous, CB– = acyanophilous, L = mean spore length (arithmetic average of all spores), W = mean spore width (arithmetic average of all spores), Q = variation in the L/W ratios between the specimens studied, *n* (a/b) = number of spores (a) measured from given number (b) of specimens. Special color terms follow Anonymous (1969) and Petersen (1996).

### Molecular sequencing

A cetyl trimethylammonium bromide rapid plant genome extraction kit (Demeter Biotechnologies Co., Ltd, Beijing) was used to extract total genomic DNA from purified isolates, and performed the polymerase chain reaction (PCR) according to the manufacturer’s instructions with some modifications (Cui et al. 2019). The DNA was amplified with the primers: ITS5/ITS4 for ITS.

(White et al. 1990), LR0R/LR7 for nLSU (Vilgalys and Hester 1990), RPB1-Af/RPB1-Cr for.

RPB1 (Matheny et al. 2002), fRPB2-5F/fRPB2-7cR for RPB2 (Liu et al. 1999; Matheny 2005), and EF1-983F/EF1-1567R for TEF1 (Rehner and Buckley 2005). The PCR procedures for ITS, nLSU, RPB1, RPB2 and TEF1 followed Shen et al. (2019) and Ji et al. (2022). DNA sequencing was performed at Beijing Genomics Institute, and the newly generated sequences were deposited in the GenBank database.

### Phylogenetic analysis

Sequences generated for this study were aligned with additional sequences downloaded from GenBank (Table 1) using BioEdit (Hall 1999) and ClustalX (Thompson et al. 1997). The data matrixes were edited in Mesquite v3.70 software (Maddison and Maddison 2021). *Trametes suaveolens* was used as outgroup. Alignment was manually adjusted to allow maximum alignment and to minimize gaps. Sequence alignment was deposited at TreeBase (http://purl.org/phylo/treebase/).

**Table 1:** Taxa information and GenBank accession numbers of the sequences used in this study

Species Name	Sample No	Locality	GenBank Accessions				
			ITS	nLSU	TEF1	RPB1	RPB2
*Antrodia serialis*	Cui 10519	China	KP715307	KP715323	KP715337	–	KR610830
*Calocera cornea*	AFTOL 438	–	AY789083	AY701526	AY881019	AY857980	AY536286
*Clavaria zollingeri*	AFTOL 563	–	AY854071	AY639882	AY881024	AH014578	AY780940
*Coltricia perennis*	AFTOL 447	–	DQ234559	AF287854	AY885147	AY864867	AY218526
*Cyanoporus* aff. *fuligo* (*Grammothele* aff. *fuligo*)	FP150657	Belize	KY948716	KY948840	–	KY948908	–
*C. camptogrammus*	Dai 19693	China	ON261649	–	OP556546	OP556530	–
*C. camptogrammus*	Dai 21948	China	ON261650	ON261621	OP556547	OP556531	OP556573
*C. camptogrammus*	Dai 22099	China	ON261651	ON261622	–	OP556532	–
*C. camptogrammus*	Dai 22117	China	ON261652	ON261623	OP556548	OP556533	–
*C. fuligo*	Dai 21117	China	ON261653	ON261624	OP556549	–	–
*C. fuligo*	Dai 21936	China	ON261654	ON261625	OP556550	OP556534	–
*C. fuligo*	Dai 21937	China	ON261655	ON261626	OP556551	–	–
*C. fuligo*	Dai 21950	China	ON261656	ON261627	OP556552	OP556535	–
*Dacryopinax spathularia*	AFTOL 454	–	AY854070	AY701525	AY881020	AY857981	AY786054
*Daedalea quercina*	Dai 12152	Czech Republic	KP171207	KP171229	KR610717	–	KR610809
*Echinodontium tinctorium*	AFTOL 455	–	AY854088	AF393056	AY885157	AY864882	AY218482
*Epithele macarangae*	FP150881	Belize	KY948713	KY948843	–	KY948909	–
*E. malaiensis*	LY 8252	Singapore	KT361636	–	–	–	–
*E. typhae*	AFTOL-ID 1724	France	DQ486701	DQ457665	–	–	–
*Fomitiporia hartigii*	MUCL 53551	Estonia	JX093789	JX093833	JX093746	–	JX093877
*F. mediterranea*	AFTOL 688	–	AY854080	AY684157	AY885149	AY864870	AY803748
*Fomitopsis betulina*	Dai 12665	Finland	KP171215	KP171238	KR610724	–	KR610817
*F. pinicola*	Cui 10405	China	KC844852	KC844857	KR610690	–	KR610781
*Grammothele denticulata*	Dai 16112	China	KU512914	–	–	–	–
*G. denticulata*	Dai 21982	China	ON261657	ON261628	OP556553	OP556536	OP556574
*G. duportii* (*G.* sp.)	Cui 6539	China	KX832049	KX832058	KX838434	KX838469	–
*G. duportii*	Dai 17821	Singapore	ON261658	ON261629	OP556554	–	–
*G. duportii*	Dai 21932	China	ON261660	ON261631	OP556555	–	OP556575
*G. hainanensis*	Cui 14514	China	OK642191	OK642246	OK665209	OK665282	–
*G. hainanensis*	Dai 16258	China	KU512915	KY475572	–	–	–
*G. lineata*	Dai 18485	Brazil	ON261661	ON261632	–	–	–
*G. lineata*	WX2014-208	Brazil	MH842147	–	–	–	–
*G. quercina*	Cui 17162	China	OK642192	OK642247	OK665210	OK665283	OK665283
*G. quercina*	Cui 17296	China	ON261662	ON261633	OP556556	OP556537	–
*G. quercina*	Cui 17714	China	OP997537	OP997546	OP556557	OP556538	–
*G.* sp.	FP150289	Jamaica	KY948717	KY948836	–	KY948904	–
*G.* sp.	FP150296	Jamaica	KY948718	–	–	–	–
*Heterobasidion annosum*	06129/6	Russia	KJ583211	KJ583225	KX252741	KF033133	KF033133
*Hygrocybe conica*	AFTOL 729	–	AY854074	AY684167	AY883425	AY860522	AY803747
*Lactarius deceptivus*	AFTOL 682	USA	AY854089	AY631899	AY885158	AY864884	AY803749
*Laetiporus montanus*	Cui 10011	China	KF951274	KF951315	KX354617	MG867670	KT894790
*L. sulphureus*	Cui 12388	China	KR187105	KX354486	KX354607	MG867671	KX354652
*Mycena aurantiidisca*	AFTOL 1685	USA	DQ490646	DQ470811	GU187728	DQ447927	DQ474122
*M. amabilissima*	AFTOL 1686	USA	DQ490644	DQ457691	GU187727	DQ447926	DQ474121
*Marasmius rotula*	AFTOL 1505	USA	DQ182506	DQ457686	GU187723	DQ447922	DQ474118
*Neurospora crassa*	CBS 367.70	–	HQ271348	AF286411	XM959775	MH871466	AF107789
*Perenniporia subtephropora*	Dai 10962	China	JQ861752	JQ861768	KF286329	KX880811	–
*P. subtephropora*	Dai 10964	China	JQ861753	JQ861769	KF286330	KX880812	–
*P. tephropora*	Cui 8040	China	JN048763	HQ654118	KF286307	KX880814	–
*P. tephropora*	Cui 9029	China	HQ876601	JF706339	–	KX880813	–
*Phaeolus schweinitzii*	Dai 8025	China	KX354457	KX354511	KX354686	–	DQ408119
*Polyporus squamosus*	AFTOL 704	–	DQ267123	AY629320	DQ028601	DQ831023	DQ408120
*Porogramme albocincta*	PR1478R	Puerto Rico	KY948724	–	–	–	–
*P. albocincta*	PR1478T	Puerto Rico	KY948725	KY948838	–	KY948906	–
*P. aurantiaca*	Dai 17401	Brazil	ON261666	ON261637	–	–	–
*P. aurantiaca* (*Grammothele aurantiaca*)	WX2014-115	Brazil	MH842137	MH844886	–	–	–
*P. austroasiana*	Dai 19624	Sri Lanka	ON261668	ON261639	–	–	–
*P. austroasiana*	Dai 19,634	Sri Lanka	ON261669	ON261640	–	–	–
*P. austroasiana*	Dai 21,202	Malaysia	ON261670	–	OP556560	–	–
*P. brasiliensis* (*Grammothele brasiliensis*)	WX2014-28	Brazil	MH844866	MH844865	–	–	–
*P. brasiliensis* (*G. brasiliensis*)	WX2014-100	Brazil	MH844679	MH844583	–	–	–
*P. bubalina* (*Tinctoporellus bubalinus*)	Yuan 5801	China	JQ319499	–	–	–	–
*P. bubalina* (*T. bubalinus*)	Yuan 5813	China	JQ319499	–	–	–	–
*P. cylindrica*	Dai 18526A	China	ON261671	ON261641	OP556561	OP556541	–
*P. cylindrica*	Dai 18529A	China	ON261672	ON261642	OP556562	OP556542	–
*P. cylindrica*	Dai 18544A	China	ON261673	ON261643	OP556563	OP556543	–
*P. cylindrica*	Dai 22,348	China	ON261674	ON261644	OP556564	OP556544	OP556576
*P. epimiltina* (*Tinctoporellus epimiltinus*)	Dai 19,483	Sri Lanka	OP997538	OP997547	OP556565	–	–
*P. epimiltina* (*T. epimiltinus*)	Dai 19,625	Sri Lanka	OP997539	OP997548	OP556566	OP556545	–
*P. hinnulea* (*T. hinnuleus*)	Dai 13,664	China	OP997540	OP997549	OP556567	–	–
*P. hinnulea* (*T. hinnuleus*)	Yuan 5832	China	JQ319500	–	–	–	–
*P. micropora* (*P. albocincta*)	FP102875sp	Puerto Rico	KY948726	–	–	–	–
*P. micropora* (*Grammothele micropora*)	WX2014-116	Brazil	MH842144	–	–	–	–
*P. subargentea*	Dai 17,445	Brazil	ON261675	ON261645	OP556568	–	–
*P. subargentea*	Dai 17,460	Brazil	ON261676	ON261646	OP556569	–	–
*P. subargentea (Grammothele subargentea)*	WX2014-26	Brazil	MH819426	MH842138	–	–	–
*P. venezuelica (G. venezuelica)*	O-F-76258	Venezuela	MT216233	–	–	–	–
*P. yunnanensis*	Dai 12,222	China	KF913423	KF913427	–	–	–
*P. yunnanensis*	Dai 12,236	China	ON261677	–	OP556570	–	–
*P. yunnanensis*	Dai 12,259	China	KF913424	KF913428	–	–	–
*Pseudogrammothele separabillima*	Dai 22,568	China	ON261664	ON261635	OP556558	OP556539	–
*P. separabillima*	Dai 22,599	China	ON261665	ON261636	OP556559	OP556540	–
*Pycnoporellus fulgens*	Cui 10,033	China	KX354458	KX354512	KX354687	–	KX354684
*Schizosaccharomyces pombe*	972 h-	–	Z19578	Z19136	NM001021161	NM001021568	NM001018498
*Sparassis crispa*	AFTOL 703	–	DQ250597	AY629321	DQ056289	–	DQ408122
*Stereum hirsutum*	AFTOL 492	–	AY854063	AF393078	AY885159	AY864886	AY218520
*Theleporus calcicolor*	Dai 7921	China	JN411117	–	–	–	
*T. calcicolor*	Dai 12,146	China	JN411118	–	–	–	
*T. membranaceus*	Dai 12,075	China	JN411120	–	–	–	
*T. membranaceus*	Dai 16,241	China	KU512920	–	–	–	
*T. minisporus*	Cui 13,623	China	ON261678	ON261647	OP556571	–	
*T. minisporus*	Dai 12011	China	JN411121	KX880675	KX880891	KX880821	
*T. venezuelicus*	ZGCVN109	India	MT876596	–	–	–	
*T. venezuelicus*	Ryvarden 35205	Venezuela	KT361631	–	–	–	
*Trametes suaveolens*	Cui 10697	China	KC848280	KC848365	KX880933	KX880839	
*T. versicolor*	FP135156sp	USA	JN164919	JN164809	JN164878	JN164825	JN164850
*Ustilago maydis*		–	AY854090	AF453938	AY885160	KP322928	KP323090
*Wolfiporia hoelen*	CBK-1	China	KX354453	KX354689	KX354688	–	KX354685

New sequences are shown in bold

Maximum Likelihood (ML) and Bayesian inference (BI) were employed to perform phylogenetic analysis using the jModeltest v.2.17 to determine the best-fit evolution model for the combined dataset of ITS + nLSU + TEF1 + RPB1 sequences for estimation. Phylogenetic analysis approaches followed Zhao et al. (2015). The ML phylogenies were inferred from the combined dataset using RAxML 7.2.8 (GitHub, San Francisco, CA, USA), the default settings of the GTR + I + G model were used for all parameters in the ML analysis (Stamatakis 2006). The ML bootstrap values (ML) of the nodes were obtained using the GTRCAT model with 1000 bootstrap replicates (Hills and Bull 1993).

The BI analyses were conducted with MrBayes 3.2.6 (Ronquist 2003). Four Markov chains were run for 2,000,000 generations until the split deviation frequency value was ≤ 0.01 and trees were sampled every 1000 generations. The first 25% of sampled trees were discarded as burn-in, whereas the remaining trees were used to construct a 50% majority consensus tree and calculate Bayesian posterior probabilities (BPPs).

Branches that received bootstrap support for BS (bootstrap support for ML) values and BPPs (Bayesian posterior probabilities for BI) simultaneously ≥ 50% and ≥ 0.8 were considered as significantly supported, respectively. Phylogenetic tree was visualized with the program FigTree v. 1.4.3 (http://tree.bio.ed.ac.uk/software/figtree/).

### Divergence time estimation

The divergence times were estimated with the BEAST v2.6.5 software package (Bouckaert et al. 2019) with a dataset composed of ITS + nLSU + TEF1 + RPB1 + RPB2 sequences (Table 1). Sequences of the species are adopted partly from the topology established by Song and Cui (2017). An XML file was generated with BEAUti (version 2). The rates of evolutionary changes at nuclear acids were estimated using ModelTest (version 3.7) with the GTR substitution model (Posada and Crandall 1998). A log-normal distribution was employed for molecular clock analysis and the tree prior was set to Yule speciation. Three fossil calibrations, *Archaeomarasmius leggettii* (Hibbett et al. 1995; 1997), *Quatsinoporites cranhamii* (Smith et al. 2004; Berbee and Taylor 2010) and *Paleopyrenomycites devonicus* (Taylor et al. 1999; 2005), representing the minimum divergence time of *Agaricales* (90 Mya), *Hymenochaetaceae* (125 Mya), and between *Ascomycota* and *Basidiomycota* (400 Mya), respectively, were used as calibrations. After 10,000,000 generations, the first 10% of the sampled trees were removed as burn-in. The log file was checked for convergence with Tracer (version 1.52), and the trees file was interpreted to a maximum clade credibility (MCC) tree with TreeAnnotator (version 2.6.5), annotating clades with more than 0.8 Bayesian posterior probability (BPP).

## Results

### Molecular phylogeny

**Fig. 1:**
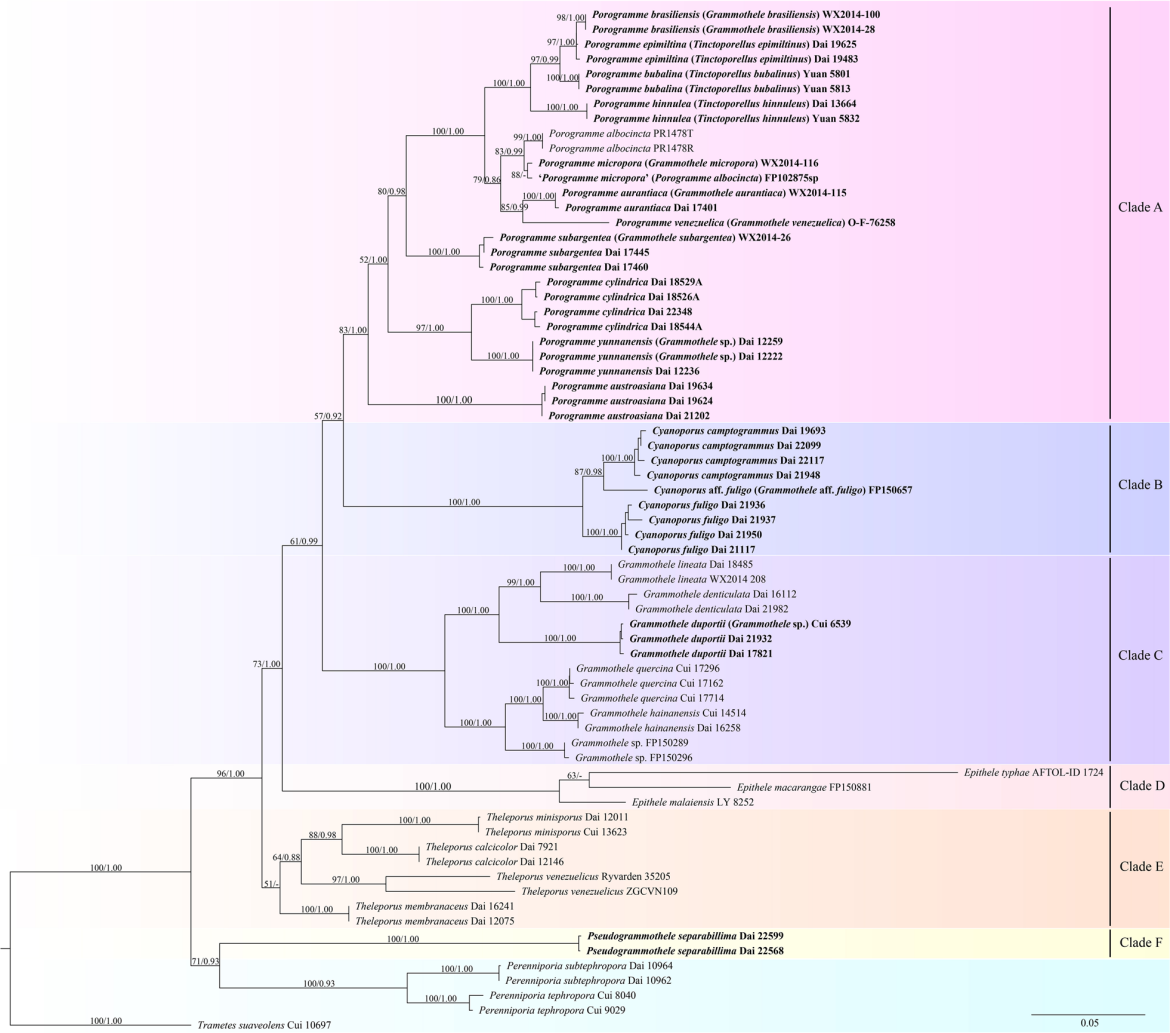
Phylogeny of *Porogramme* and related species generated by Maximum Likelihood based on dataset ITS + nLSU + TEF1 + RPB1. Bootstrap supports for Maximum Likelihood (ML) and Bayesian Posterior Probabilities (BPPs) are higher than 50% (ML) and 0.8 (BPPs) on the branches. New taxa are in bold

The combined dataset included sequences from 69 fungal specimens representing 32 taxa. Best model for the combined ITS + nLSU + TEF1 + RPB1 dataset estimated and applied in the Bayesian analysis: GTR + I + G, lset nst = 6, rates = invgamma; prset statefreqpr = dirichlet (1, 1, 1, 1). Bayesian analysis resulted in an average standard deviation of split frequencies = 0.003213. Both ML and BI trees resulted in similar topologies, thus only the topology from the ML analysis is presented along with statistical values from the ML (≥ 50%) and BPPs (≥ 0.8) algorithms (Fig. 1). From the phylogenetic tree, four new well-supported lineages in *Porogramme* clade (Clade A) were formed: Three specimens (Dai 19624, 19634 and 21202) from Malaysia, and Sri Lanka named as *Porogramme austroasiana*; three specimens (Dai 12222, 12236 and 12259) from Yunnan of China named as *P. yunnanensis*; four samples (Dai 18526A, 18529A, 18544A and 22348) from subtropical China named as *P. cylindrica;* three samples (Dai 17445, 17460 and WX2014-26) from Brazil represent *P. subargentea.*

Samples of so-called *Grammothele fuligo* formed a clade (Clade B) closely related to *Porogramme*, *Grammothele, Epithele*, and *Theleporus*. So, a new genus, *Cyanoporus*, is set up for the clade. These samples formed three lineages nested in the clade representing *C. camptogrammus*, *C. fuligo*, and *C.* aff. *fuligo*, respectively.

Three samples of Cui 6539, Dai 17821 and 21932 from southern China and Singapore formed a new lineage in the *Grammothele* clade (Clade C) with a robust support (100% ML and 1.00 BPP), and they represent *G. duportii.* Two samples of *Grammothele separabillima* H.S. Yuan from southern China formed a clade (Clade F) with a robust support (100% ML and 1.00 BPP) in our phylogeny (Fig. 1), thus the new genus, *Pseudogrammothele*, is established to accommodate the unique species.

### Divergence time estimation of *Porogramme* and related genera

**Fig. 2:**
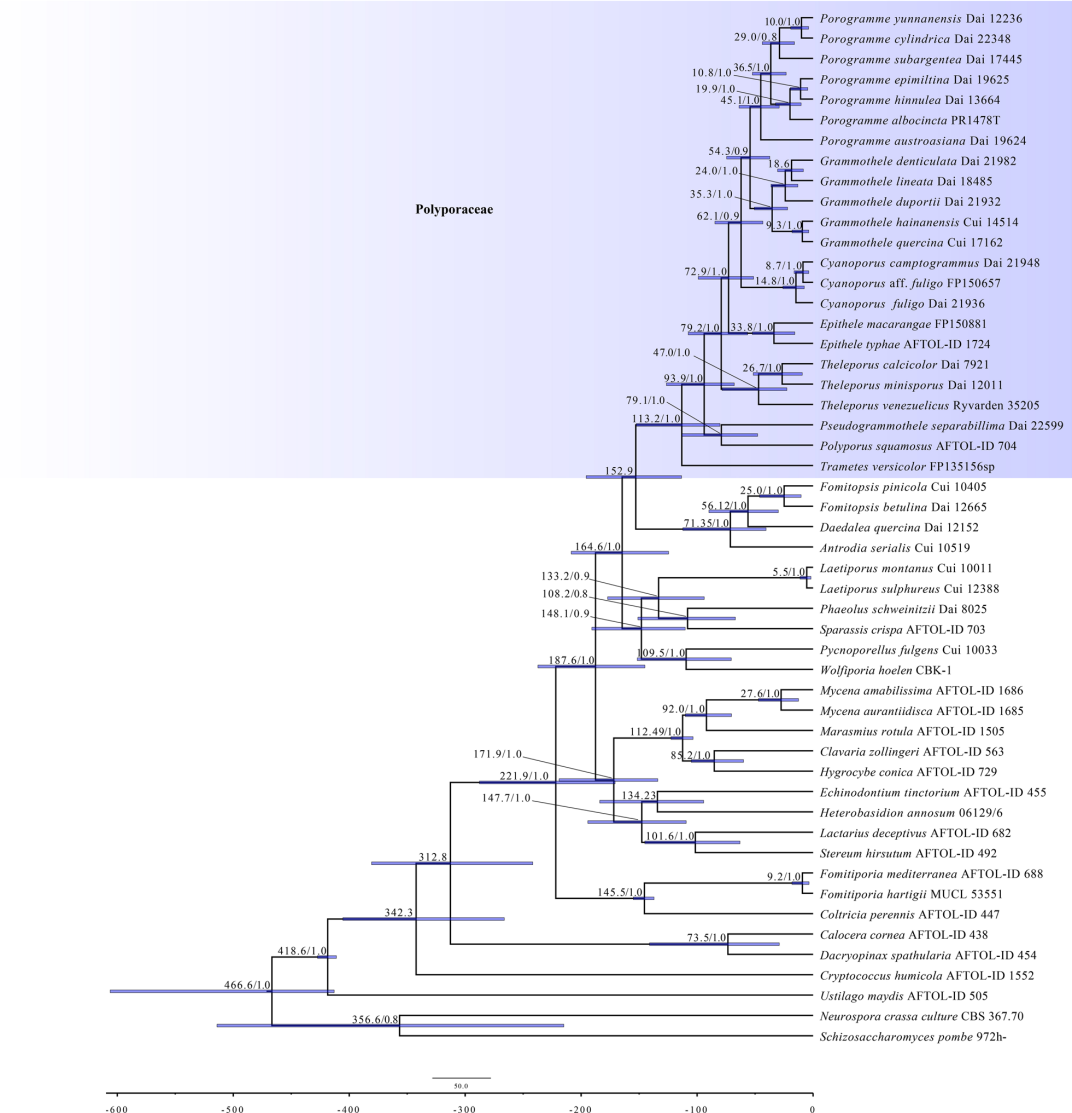
Estimated divergence times of *Porogramme* and related genera generated by molecular clock analyses using a combined dataset ITS + nLSU + TEF1 + RPB1 + RPB2. Estimated mean divergence time (Mya) and posterior probabilities (PP) > 0.8 are annotated at the internodes. The 95% highest posterior density (HPD) interval of divergence time estimates is marked by horizontal blue bars

The combined dataset for the molecular clock analysis included 51 taxa, of which 33 belonged to *Polyporaceae*. The results of divergence time estimation (Fig. 2) show that *Polyporaceae* emerged in a mean stem age of 152.9 Mya [95% highest posterior density (HPD) of 113.4–195.6 Mya] and a mean crown age of 113.2 Mya (95% HPD of 80.3–152.4 Mya). Among the six clades of *Porogramme* and related genera, *Pseudogrammothele separabillima* grouped with *Polyporus squamosus* and evolved from the same ancester dated to 79.1 Mya. The *Theleporus* clade and *Epithele* clade diverged at 79.2 Mya and 72.9 Mya, respectively. Then, the *Cyanoporus* clade, species growing only on monocotyledons which previously treated as *Grammothele fuligo*, diverged at 62.1 Mya. The *Porogramme* clade, including the species of *Porogramme* and *Tinctoporellus*, sister to the *Grammothele* clade, and both of them diverged at 54.3 Mya. Consequently, the mean stem ages of the six major clades are well supported as allied lineages originated during the middle Paleogene period which is consistent with previous studies of Ji et al. (2022).

### Taxonomy

**Fig. 3:**
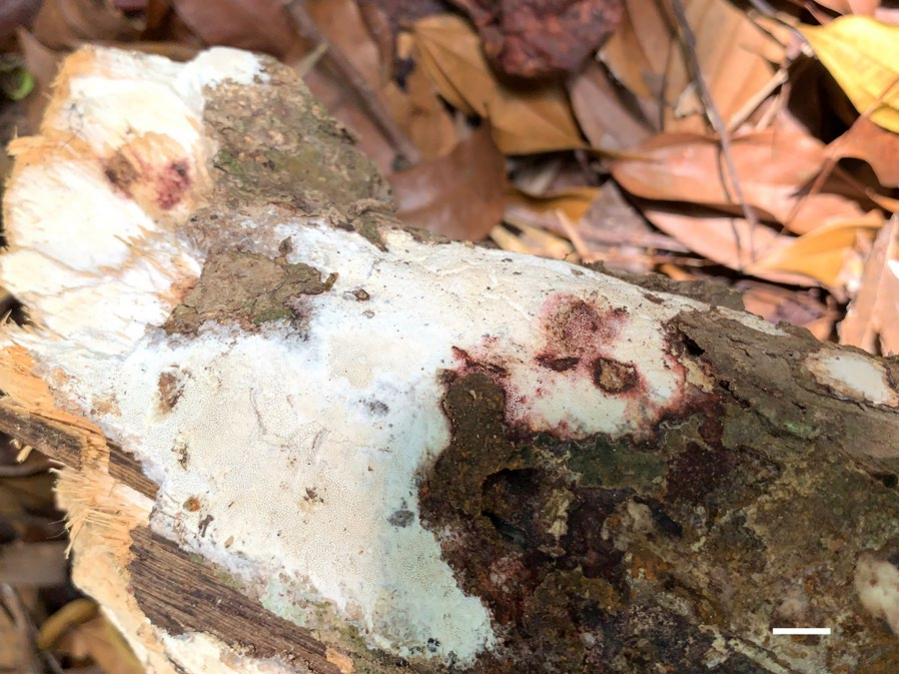
Basidiome of *Porogramme austroasiana* (holotype). Bar = 1.0 cm

**Fig. 4:**
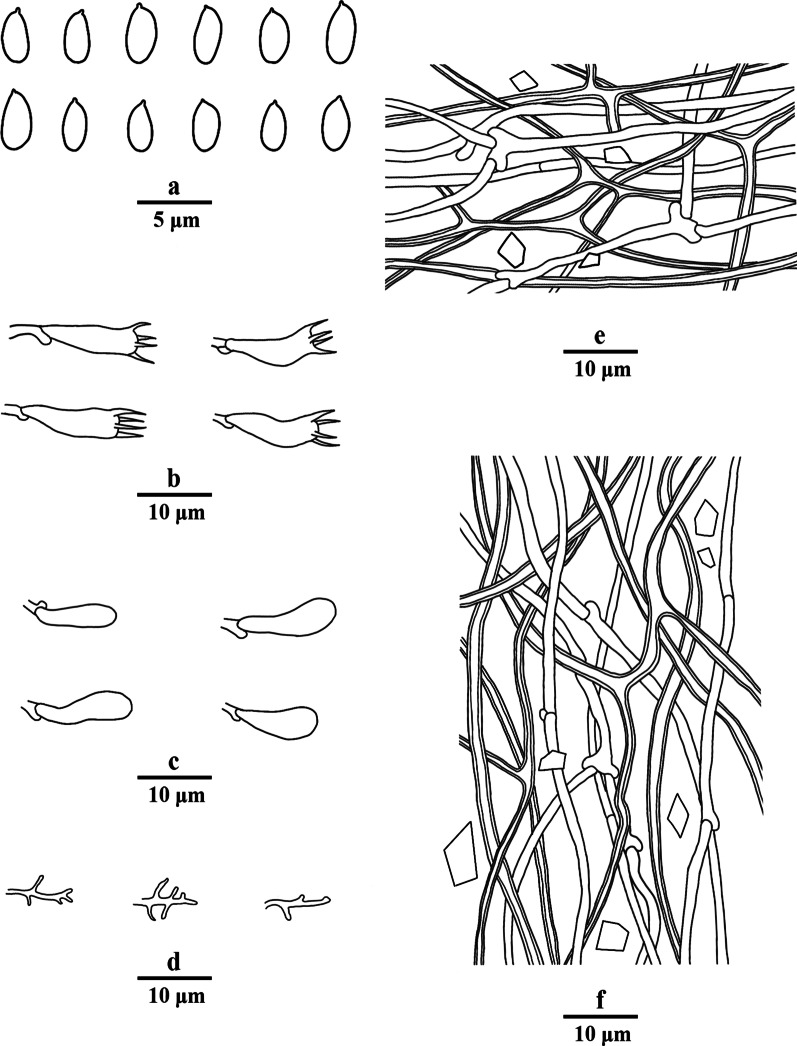
Microscopic structures of *Porogramme austroasiana* (drawn from holotype). a. Basidiospores; b. Basidia; c. Basidioles; d. Dendrohyphidia; e. Hyphae from subiculum; f. Hyphae from trama

*Porogramme austroasiana* Y.C. Dai, W.L. Mao & Yuan Yuan, sp. nov., (Figs. 3 and 4).

MycoBank no.: MB 846852.

*Etymology: Austroasiana* (Lat.): refers to the species being found in South Asia.

*Diagnosis:* The irregular and partly split pores, thin-walled generative hyphae with both clamps and simple septa, arboriform branched skeletal hyphae, clavate basidia sometimes constricted at middle, oblong ellipsoid to narrowly ovoid basidiospores measuring 3.8–4.5 × 2.1–2.5 μm differentiate the species in *Porogramme*.

*Type:* Sri Lanka: Avissawella, Salgala Forest, N 6° 95′, E 80° 20′, on fallen angiosperm trunk, 3 March 2019, *Y.C. Dai 19624* (BJFC031301—holotype).

*Description: Basidiome* annual, resupinate, difficult to separate from the substrate, hard and brittle when dry, to 7.5 cm long, 4.5 cm wide and 0.6 mm thick at the center. Pore surface cream to pale buff when fresh and dry; sterile margin distinct, white when fresh, cream when dry; pores round to angular, irregular and partly split, 2–5 per mm; dissepiments thin, entire. Hymenium present at both vertical tube-walls and the base of tubes. *Subiculum* pale buff, resinous, to 0.1 mm thick. *Tubes* concolorous with pore surface, corky, to 0.5 mm long. *Hyphal system* dimitic; generative hyphae with both clamp connections and simple septa; skeletal hyphae IKI–, CB–; tissues unchanged in KOH. *Subicular* generative hyphae frequent, hyaline, thin-walled, moderately branched, 1.5–2.5 µm diam; skeletal hyphae dominant, pale yellowish, thick-walled with a narrow to medium lumen, frequently arboriform branched, flexuous, interwoven, 1–2.5 µm diam. *Tramal* generative hyphae frequent, hyaline, thin-walled, occasionally branched, 1.5–2 µm diam; skeletal hyphae dominant, pale yellowish, distinctly thick-walled with a medium to wide lumen, arboriform branched, flexuous, interwoven, 1.5–2.5 µm diam. *Cystidia and cystidioles* absent. *Dendrohyphidia* hyaline, thin-walled. *Basidia* clavate, sometimes constricted at middle, with 4 sterigmata and a basal clamp connection, 11–15 × 3–5 µm; basidioles dominant, clavate to pyriform, slightly smaller than basidia. Some irregular-shaped crystals present among hymenium. *Basidiospores* oblong ellipsoid to narrowly ovoid, hyaline, thin-walled, smooth, IKI–, CB–, (3.6–)3.8–4.5(–5) × (2.0–)2.1–2.5(–2.6) µm, L = 4.11 µm, W = 2.22 µm, Q = 1.82–1.85 (*n* = 60/2).

*Additional specimens examined:* Malaysia**:** *Selangor*: Kota Damansara, Community Forest Reserve, N 3° 16′, E 101° 58′, on fallen angiosperm trunk, 7 December 2019, *Y.C. Dai 21202* (BJFC032856). Singapore: Bukit Timah Nature Reserve, N 1° 35′, E 103° 77′, on fallen angiosperm trunk, 19 July 2017, *Y.C. Dai 17817* (BJFC025349). Sri Lanka: *Avissawella*: Salgala Forest, N 6° 95′, E 80° 20′, on fallen angiosperm trunk, 3 March 2019, *Y.C. Dai 19634* (BJFC031311).

**Fig. 5:**
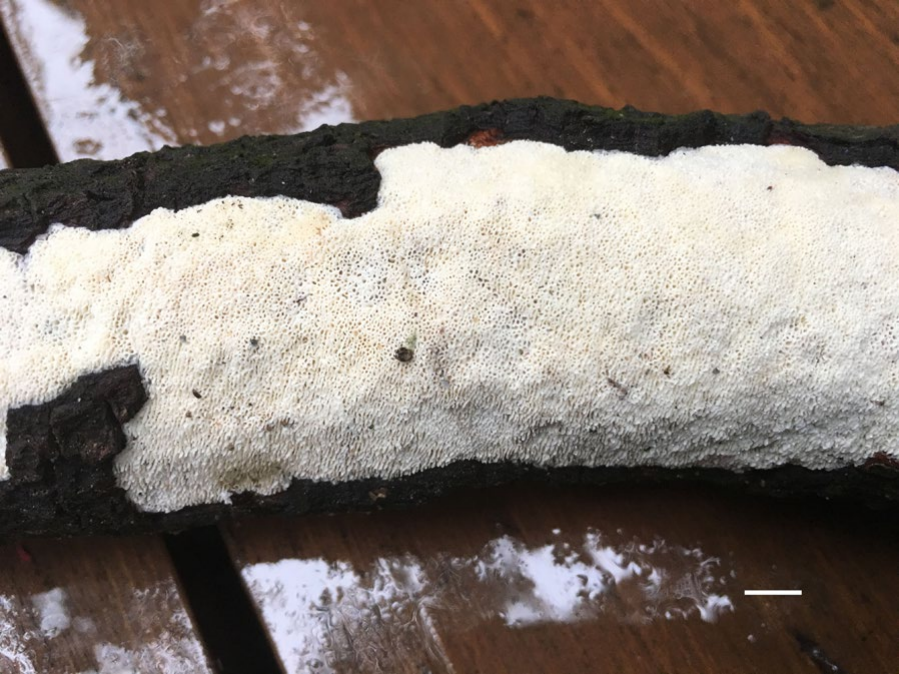
Basidiome of *Porogramme cylindrica* (holotype). Bar = 1.0 cm

**Fig. 6:**
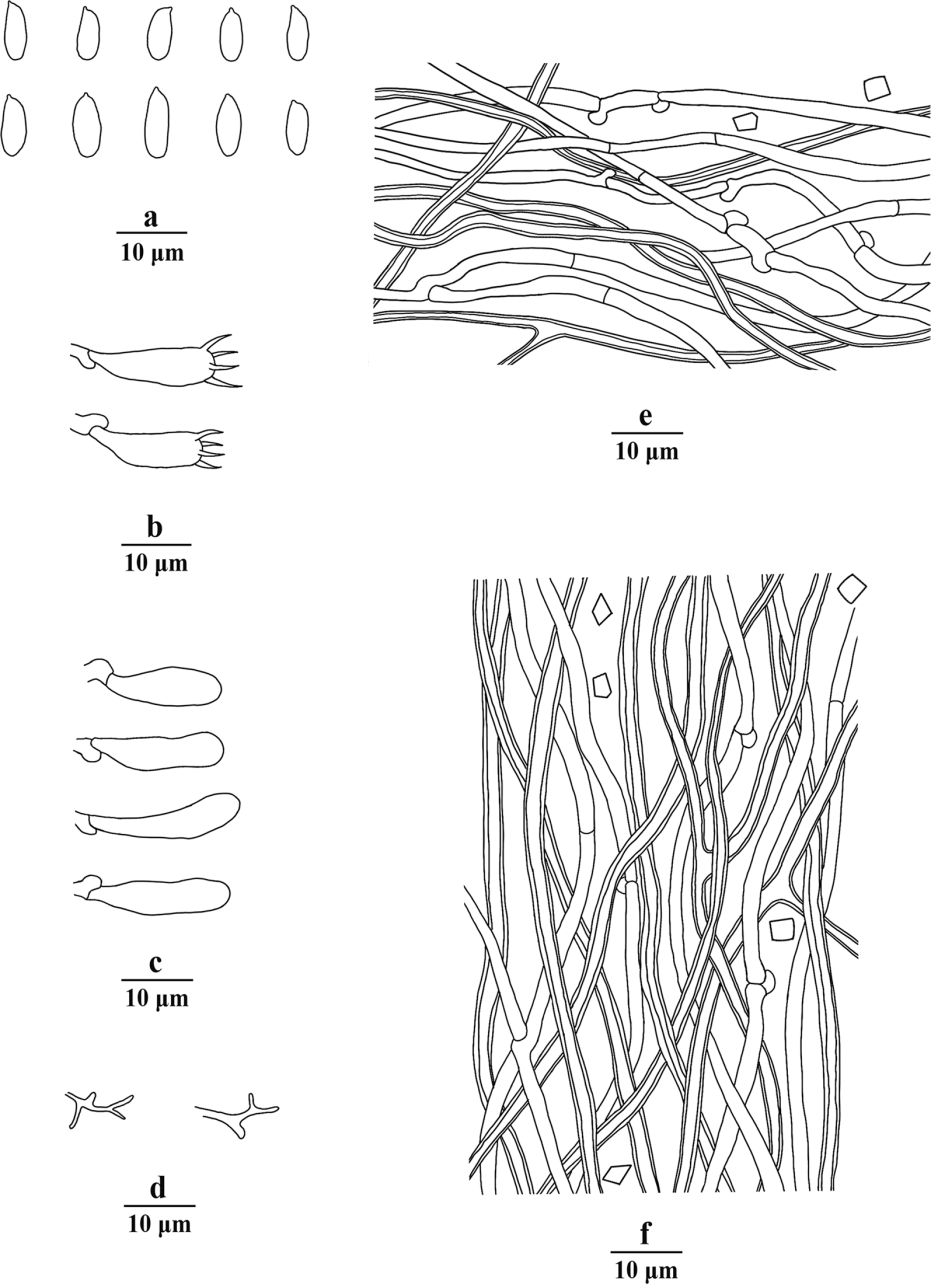
Microscopic structures of *Porogramme cylindrica* (drawn from holotype). a. Basidiospores; b. Basidia; c. Basidioles; d. Dendrohyphidia; e. Hyphae from subiculum; f. Hyphae from trama

*Porogramme cylindrica* Y.C. Dai, W.L. Mao & Yuan Yuan, sp. nov., (Figs. 5 and 6).

MycoBank no.: MB 846851.

*Etymology: Cylindrica* (Lat.): refers to the species having cylindrical basidiospores.

*Diagnosis:* Differs from other *Porogramme* species by the big pores measuring 2–4 per mm, generative hyphae with both clamp connections and simple septa, non-dextrinoid skeletal hyphae, and the cylindrical basidiospores measuring 8–10 × 3.1–3.8 µm.

*Type:* China: *Guangdong:* Zhaoqing, Dinghushan Nature Reserve, N 23° 17′, E 112° 54′, on fallen branch of *Schima*, 28 April 2018, *Y.C. Dai 18544A* (BJFC027012—holotype).

*Description: Basidiome* annual, resupinate, inseparable, corky when fresh, corky to brittle when dry, to 5 cm long, 1.8 cm wide, and 0.8 mm thick at center; sterile margin thinning out, very narrow to almost lacking. Pore surface white when fresh, straw yellow when dry; pores angular, 2–4 per mm; dissepiments thin, entire to slightly lacerate. *Subiculum* white, corky, to 0.1 mm thick. *Tubes* concolorous with pore surface, corky, to 0.7 mm long. *Hyphal system* dimitic; generative hyphae with both clamp connections and simple septa; skeletal hyphae IKI–, CB + ; tissues unchanged in KOH. *Subicular* generative hyphae dominant, hyaline, thin-walled, occasionally branched, 2–3 µm diam; skeletal hyphae frequent, hyaline, thick-walled with a narrow to medium lumen, moderately branched, flexuous, interwoven, 2–3 µm diam. *Tramal* generative hyphae frequent, hyaline, thin-walled, occasionally branched, 1.5–2 µm diam; skeletal hyphae dominant, hyaline, thick-walled with a medium to wide lumen, moderately branched, flexuous, interwoven, 1.5–3 µm diam. *Cystidia and cystidioles* absent*. Dendrohyphidia* frequent at dissepiment edges. *Hyphal pegs* occasionally present at dissepiment edge. *Basidia* clavate, with four sterigmata and a basal clamp connection, 19–23 × 4.5–7 µm; basidioles in shape similar to basidia, but slightly smaller. Some irregular-shaped crystals frequently present among hymenium. *Basidiospores* cylindrical tapering at apiculus, hyaline, thin-walled, smooth, IKI–, CB–, (7.5–)8–10(–10.2) × (3–)3.1–3.8(–4.2) µm, L = 8.99 µm, W = 3.31 µm, Q = 2.72–2.75 (*n* = 60/2).

*Additional specimens examined*: China: *Fujian:* Fuzhou, Fuzhou National Forest Park, N 26° 16′, E 119° 29′, on fallen angiosperm branch, 4 June 2021, *Y.C. Dai 22348* (BJFC036936). *Guangdong:* Zhaoqing, Dinghushan Nature Reserve, N 23° 17′, E 112° 54′, on fallen branch of *Schima*, 28 April 2018, *Y.C. Dai 18526A* (BJFC026994), *18529A* (BJFC026997).

**Fig. 7:**
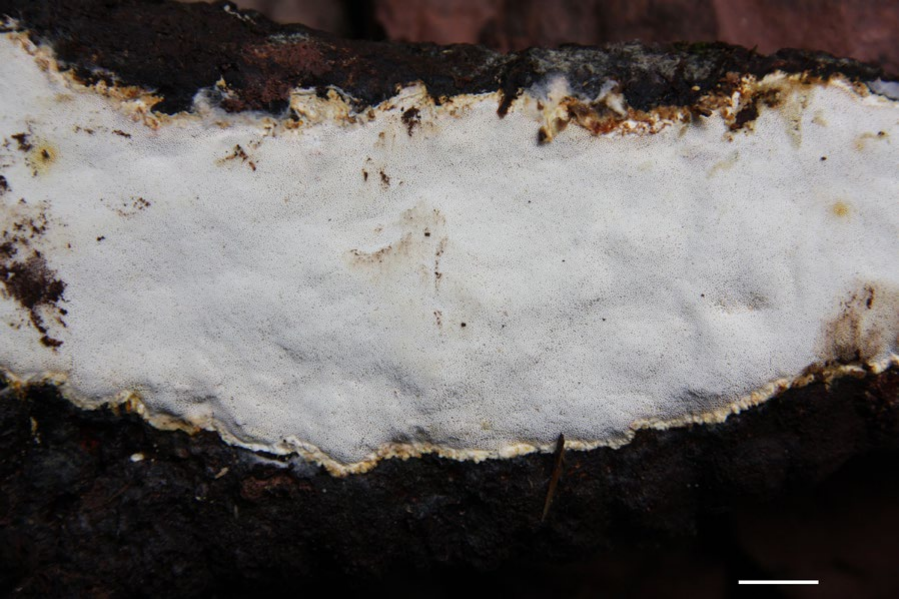
Basidiome of *Porogramme yunnanensis* (holotype). Bar = 1.0 cm

**Fig. 8:**
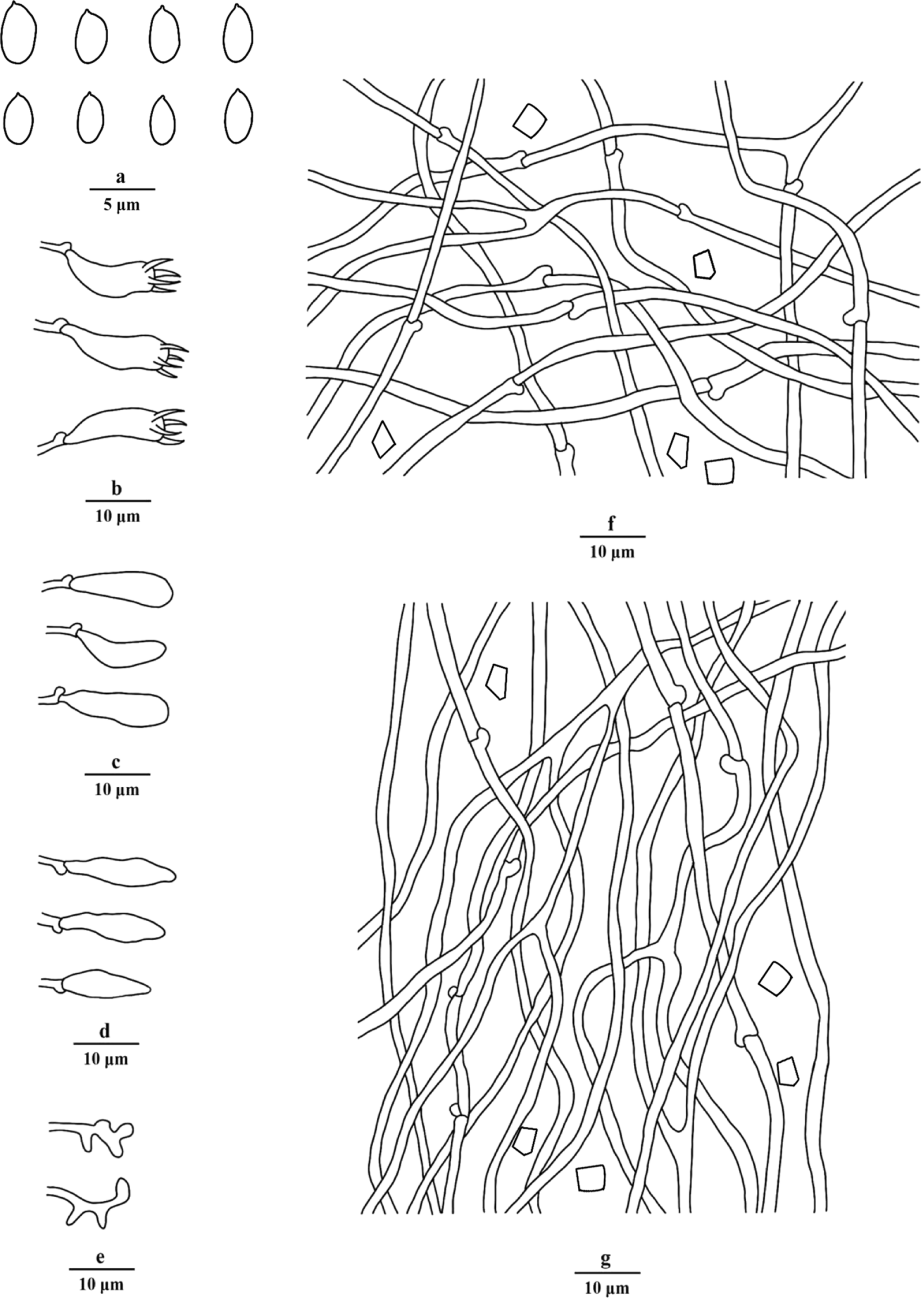
Microscopic structures of *Porogramme yunnanensis* (drawn from holotype). a. Basidiospores; b. Basidia; c. Basidioles; d. Cystidioles; e. Dendrohyphidia; f. Hyphae from subiculum; g. Hyphae from trama

*Porogramme yunnanensis* Y.C. Dai, W.L. Mao & Yuan Yuan, sp. nov., (Figs. 7 and 8).

MycoBank no.: MB 846850.

*Etymology: Yunnanensis* (Lat.): refers to the species being found in Yunnan, China.

*Diagnosis: Porogramme yunnanensis* is characterized by annual to biennial and resupinate basidiome, white pores when fresh, yellowish when dry, entire dissepiments, a monomitic hyphal system, tissues almost unchanged in KOH, the presence of fusiform cystidioles and abundant dendrohyphidia, ellipsoid to drop-shaped basidiospores measuring 3.7–5 × 2–2.6 µm.

*Type:* China: *Yunnan:* Pu'er, Laiyang River Forest Park, N 22° 78′, E 100° 97′, on fallen angiosperm trunk, 6 June 2011, *Y.C. Dai 12222* (BJFC010505 – holotype).

*Description: Basidiome* annual to biennial, resupinate, inseparable, corky and without odor or taste when fresh, becoming hard corky upon drying, to 7 cm long, 3 cm wide, and 1.6 mm thick at center; sterile margin distinct, yellowish brown when fresh. Pore surface pure white when fresh, honey when dry; pores round to angular, 4–6 per mm; dissepiments thick, entire. Hymenium present at both the vertical tube-walls and the base of tubes. *Subiculum* pale buff, resinous, to 1 mm thick. *Tubes* concolorous with the pore surface, corky, to 0.6 mm long. Wood reddening under basidiome. *Hyphal system* monomitic; generative hyphae with clamp connections, CB + ; tissues unchanged in KOH. *Subicular* generative hyphae hyaline, thin-walled, moderately branched, interwoven, 1.5–2.5 µm diam. *Tramal* generative hyphae hyaline, thin-walled, frequently branched, interwoven, 1–2.5 µm diam. *Dendrohyphidia* present in hymenium. *Cystidioles* fusiform, thin-walled, smooth, 11–15 × 2.5–4 mm. *Basidia* clavate, with four sterigmata and a basal clamp connection, 13–16.0 × 4–5.5 µm; basidioles in shape similar to basidia, but smaller. Small tetrahedric or polyhedric crystals frequently present among hymenium. *Basidiospores* ellipsoid tapering to apiculus, hyaline, thin-walled, smooth, IKI–, CB–, (3.2–)3.7–5.0(–5.1) × (1.9–)2.0–2.6(–3.0) µm, L = 4.20 µm, W = 2.31 µm, Q = 1.82–1.85 (*n* = 60/2).

*Additional specimens examined: *China: *Yunnan:* Pu'er, Laiyang River Forest Park, N 22° 78′, E 100° 97′, on fallen angiosperm trunk, 6 June 2011, *Y.C. Dai 12236* (BJFC010519), *12259* (BJFC010542), *12261* (BJFC010544).

The following eight taxa nested in the *Porogramme* clade, and their combinations are proposed:

*Porogramme aurantiaca* (A.M.S. Soares) Y.C. Dai, W.L. Mao & Yuan Yuan, comb. nov.

MycoBank no.: MB 846848.

*Basionym: Grammothele aurantiaca* A.M.S. Soares, *Fungal Divers.* 96: 212 (2019).

*Porogramme brasiliensis* (Ryvarden) Y.C. Dai, W.L. Mao & Yuan Yuan, comb. nov.

MycoBank no.: MB 846846.

*Basionym: Grammothele brasiliensis* Ryvarden, *Syn. Fung.* 33: 38 (2015); as '*brasilensis*'.

*Porogramme bubalina* (H.S. Yuan) Y.C. Dai, W.L. Mao & Yuan Yuan, comb. nov.

MycoBank no.: MB 846845.

*Basionym: Tinctoporellus bubalinus* H.S. Yuan, *Mycol. Prog.* 11: 949 (2012).

*Porogramme epimiltina* (Berk. & Broome) Y.C. Dai, W.L. Mao & Yuan Yuan, comb. nov.

MycoBank no.: MB 846844.

*Basionym: Polyporus epimiltinus* Berk. & Broome, *J. Linn. Soc., Bot.* 14: 54 (1875).

*Synonym: Tinctoporellus epimiltinus* (Berk. & Broome) Ryvarden, *Trans. Br. mycol. Soc.* 73: 18 (1979).

*Porogramme hinnulea* (H.S. Yuan) Y.C. Dai, W.L. Mao & Yuan Yuan, comb. nov.

MycoBank no.: MB 846843.

*Basionym: Tinctoporellus hinnuleus* H.S. Yuan, *Mycol. Prog.* 11: 950 (2012).

*Porogramme micropora* (A.M.S. Soares & W.K.S. Xavier) Y.C. Dai, W.L. Mao & Yuan Yuan, comb. nov.

MycoBank no.: MB 846841.

*Basionym: Grammothele micropora* A.M.S. Soares & W.K.S. Xavier, *Fungal Divers.* 96: 212 (2019).

*Porogramme subargentea* (Speg.) Y.C. Dai, W.L. Mao & Yuan Yuan, comb. nov.

MycoBank no.: MB 846840.

*Basionym: Poria subargentea* Speg., *Revista Argent. Hist. Nat.* 1: 104 (1891).

*Synonym: Grammothele subargentea* (Speg.) Rajchenb., *Mycotaxon* 17: 280 (1983).

*Material examined:* Brazil: *Pernambuco:* Recife, Charles Darwin Ecological Reserve, S 8° 40′, W 34° 52′, on fallen angiosperm trunk, 18 May 2017, *Y.C. Dai 17445* (BJFC024976), *17460* (BJFC024991).

*Remarks* *:* *Porogramme subargentea.*

was originally described as *Poria subargentea* from South America (Spegazzini 1891). Then Rajchenberg (1983) combined it into *Grammothele* according to its dextrinoid skeletal hyphae, abundant dendrohyphidia and cylindric basidiospores. Afterwards, Reck and Silveira (2009) found its hymenium covers the vertical tube walls and the substrate with reddish zones. These features are similar to *Porogramme epimiltina*, but phylogenetically it is closer to *P. cylindrica* rather than *P. epimiltina* (Fig. 1).

*Porogramme venezuelica* (Ryvarden) Y.C. Dai, W.L. Mao & Yuan Yuan, comb. nov.

MycoBank no.: MB 846839.

*Basionym: Grammothele venezuelica* Ryvarden, *Syn. Fung.* 33: 42 (2015).

Based on our study, *Tinctoporellus* merged in *Porogramme*, and some species previously addressed in *Grammothele* are combined in *Porogramme*. We definite *Porogramme* as following.

*Basidiome* resupinate. *Hymenophore* cream, bluish gray, reddish to almost black, irpicoid to poroid. *Hymenium* present at both the vertical tube-walls and the base of tubes or restricted to the base of tubes. *Hyphal system* monomitic or dimitic, generative hyphae with clamp connections or with both clamp connections and simple septa, hyphae dextrinoid or not. *Cystidia* absent. *Dendrohyphidia* present in most species. *Basidiospores* ellipsoid to cylindrical, thin-walled, IKI–, CB–*. Ecology* a white rot and reddening substrate in most species.

*Cyanoporus* Y.C. Dai, W.L. Mao & Yuan Yuan, gen. nov.

MycoBank no.: MB 846831.

*Etymology: Cyanoporus* (Lat.): refers to the genus having bluish pores.

*Type: Cyanoporus fuligo* (Berk. & Broome) Y.C. Dai, W.L. Mao & Yuan Yuan.

*Description: Basidiome* annual, resupinate, adnate, corky to coriaceous. Pore surface bluish gray to dark blue. *Hyphal system* dimitic; generative hyphae with clamp connections; skeletal hyphae IKI–, CB–. Hymenium restricted to the base of tubes. *Basidiospores* ellipsoid, hyaline, thin-walled, smooth, IKI–, CB–. Causing a white rot, usually growing on monocotyledons.

**Fig. 9:**
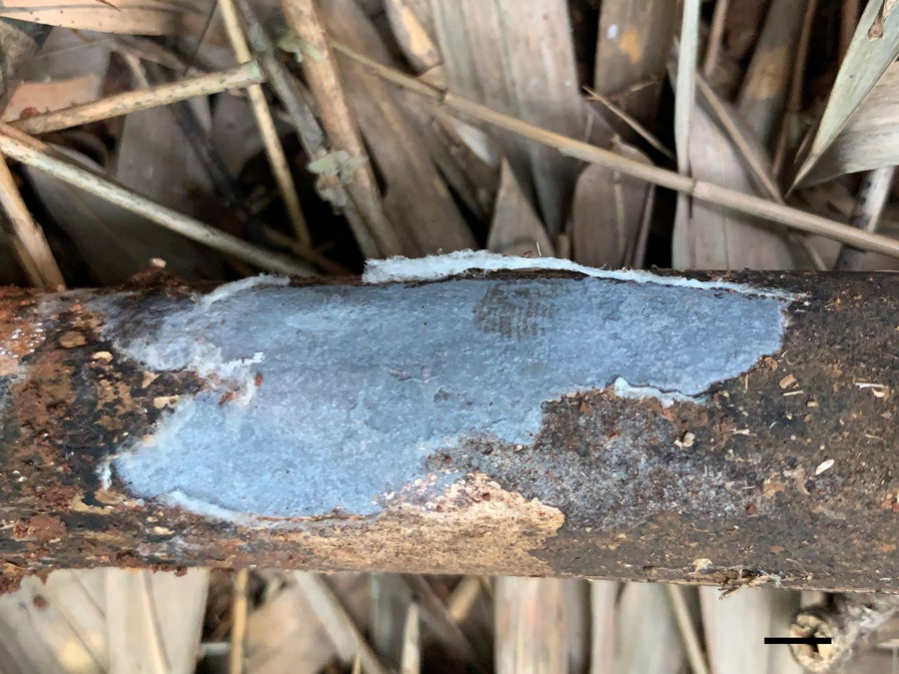
Basidiome of *Cyanoporus fuligo* (*Y.C. Dai 21936*). Bar = 1.0 cm

**Fig. 10:**
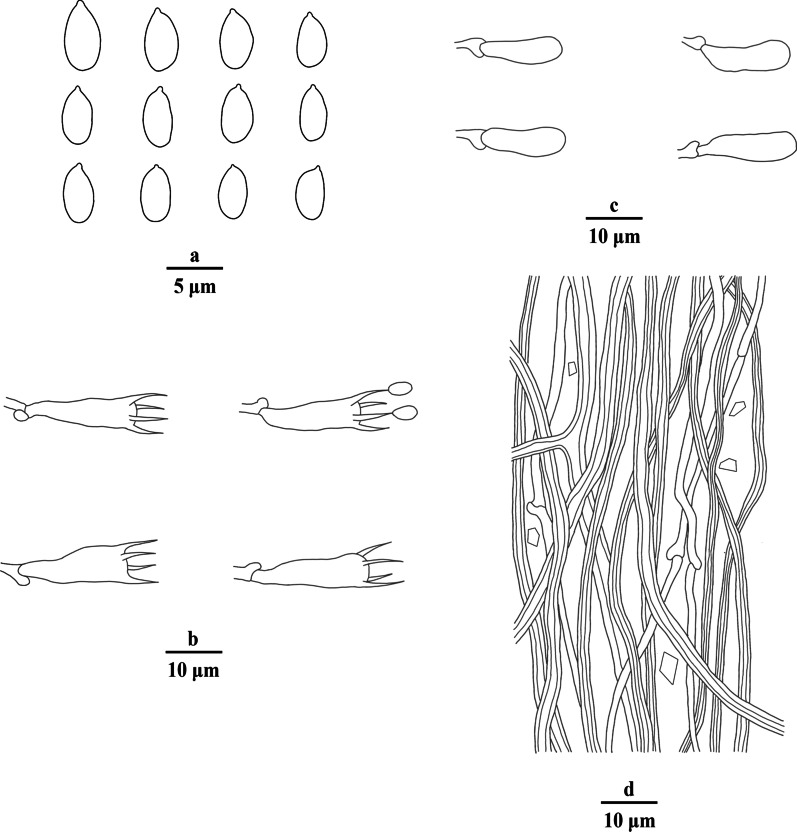
Microscopic structures of *Cyanoporus fuligo* (*Y.C. Dai 21936*). a. Basidiospores; b. Basidia; c. Basidioles; d. Hyphae from trama

*Cyanoporus fuligo* (Berk. & Broome) Y.C. Dai, W.L. Mao & Yuan Yuan, comb. nov., (Figs. 9 and 10).

MycoBank no.: MB 846834.

*Basionym: Polyporus fuligo* Berk. & Broome, *Bot. J. Linn. Soc.* 14: 53 (1875).

*Synonym: Grammothele fuligo* (Berk. & Broome) Ryvarden, *Trans. Br. mycol. Soc.* 73: 15 (1979).

*Description: Basidiome* annual, resupinate, adnate, inseparable, leathery to corky when fresh, corky when dry; to 13 cm, 2.5 cm wide and 0.3 mm thick at center. Pore surface bright grayish blue when fresh, becoming dark blue upon drying; pores angular, 8–12 per mm; dissepiments thin, entire. Sterile margin thinning out, pale bluish gray, to 1 mm wide. Hymenium restricted to the base of tubes. *Subiculum* dark brown, hard corky, very thin, to 0.04 mm thick. *Tubes* hard corky, to 0.26 mm long, tube walls whitish under a lens, but trama dark brown. *Hyphal system* dimitic; generative hyphae with clamp connections; skeletal hyphae IKI–, CB–; tissues becoming pale olivaceous to dark brown in KOH. *Subicular* hyphae is similar to those in trama. *Tramal* generative hyphae infrequent, hyaline, thin-walled, occasionally branched, 1.5–2.5 μm diam; skeletal hyphae dominant, pale to dark brown, thick-walled with a narrow lumen to subsolid, occasionally branched, subparallel along the tubes to loosely interwoven, 1.5–3 μm diam. *Cystidia and cystidioles* absent. *Dendrohyphidia* not seen. *Basidia* clavate, with four large sterigmata and a basal clamp connection, 19–23 × 4–6 μm; basidioles in shape similar to basidia, but smaller. Some irregular-shaped crystals present among hymenium. *Basidiospores* ellipsoid, hyaline, thin-walled, smooth, IKI–, CB–, (4.2–)4.5–6(–6.8) × (2.3–)2.6–3.2(–3.5) μm, L = 5.15 μm, W = 2.91 μm, Q = 1.76–1.77 (*n* = 60/2).

*Specimens examined:* China: *Hainan:* Haikou, Jinniuling Park, N 20° 01′, E 110° 32′, on dead bamboo, 7 November 2020, *Y.C. Dai 21936* (BJFC035835), *21937* (BJFC035836), *21950* (BJFC035848). *Hunan:* Yongzhou, Xiaoxiang Park, 26° 49′, E 111° 58′, on dead bamboo, 3 November 2019, *Y.C. Dai* *21117* (BJFC032777).

**Fig. 11:**
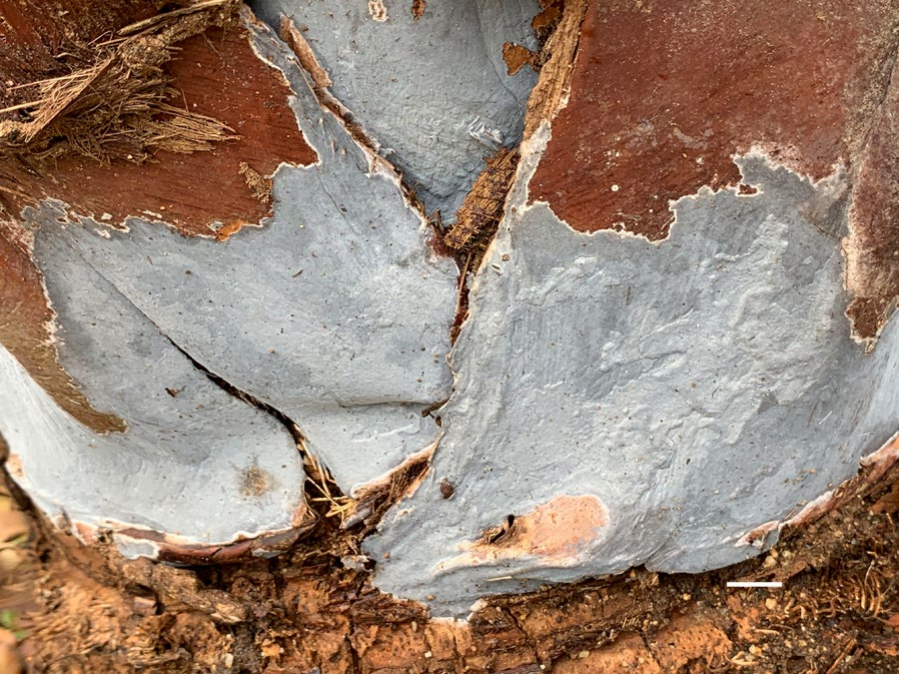
Basidiome of *Cyanoporus camptogrammus* (*Y.C. Dai 22099*). Bar = 1.0 cm

**Fig. 12:**
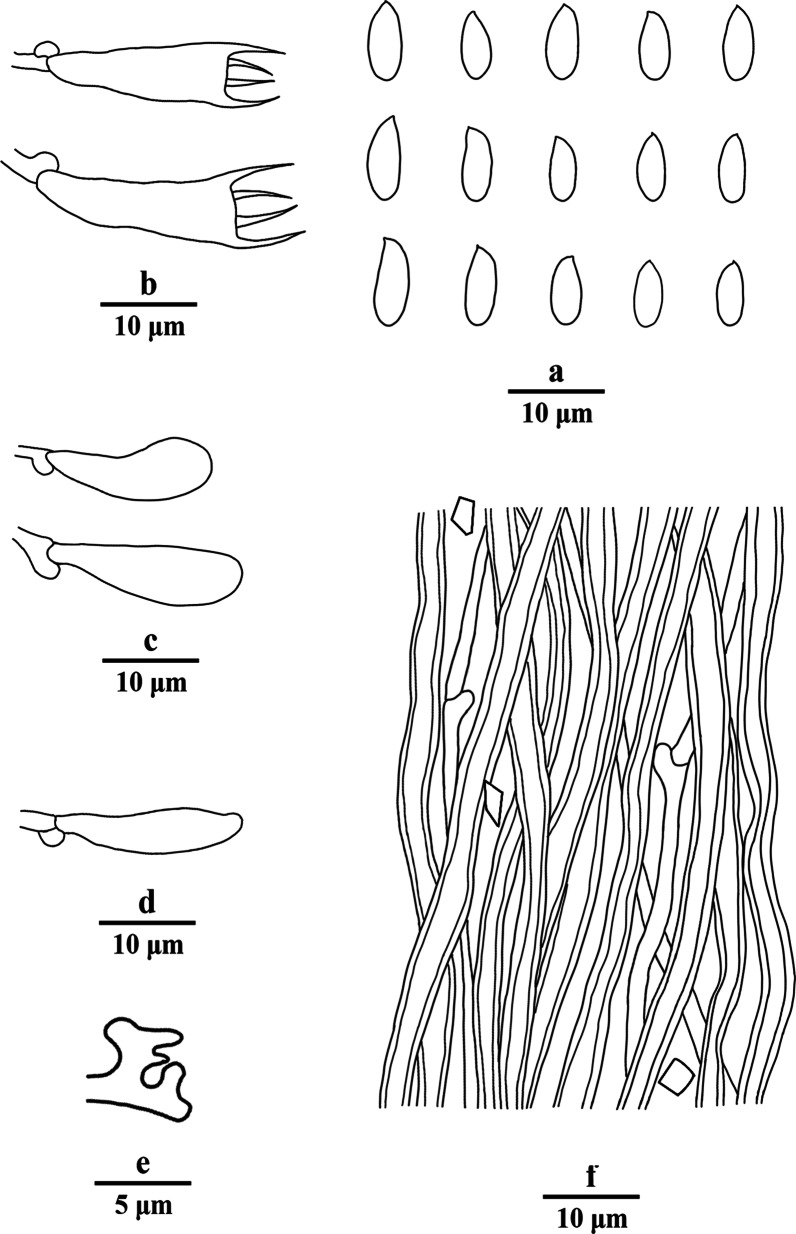
Microscopic structures of *Cyanoporus camptogrammus* (*Y.C. Dai 22099*). a. Basidiospores; b. Basidia; c. Basidioles; d. A cystidiole; e. A dendrohyphidium; f. Agglutinated skeletal hyphae; g. Hyphae from trama

*Cyanoporus camptogrammus* (Pat.) Y.C. Dai, W.L. Mao & Yuan Yuan, comb. nov., (Figs. 11 and 12).

MycoBank no.: MB 846835.

*Basionym: Porogramme camptogramma* Pat., *Bull. Soc. mycol. Fr.* 29: 208 (1913).

*Description: Basidiome* annual, resupinate, adnate, inseparable, leathery to corky; to 13 cm, 8 cm wide and 0.2 mm thick at center. Pore surface grayish blue when fresh, darkening upon drying; pores angular, 5–8 per mm; dissepiments thin, entire. Sterile margin wide to narrow, white, to 1 mm wide. Hymenium restricted to the base of tubes. *Subiculum* dark brown, corky, very thin, to 0.05 mm thick. *Tubes* corky, to 0.15 mm long, tube walls bluish white under a lens, but trama dark brown. *Hyphal system* dimitic; generative hyphae with clamp connections; skeletal hyphae IKI–, CB–; tissues becoming olivaceous to dark in KOH. *Subicular* hyphae is similar to those in trama. *Tramal* generative hyphae infrequent, hyaline, thin-walled, occasionally branched, 2–2.5 μm diam; skeletal hyphae dominant, as brown bundles of strongly agglutinated hyphae, thick-walled with a wide lumen, rarely branched, subparallel along the tubes to loosely interwoven, 2.5–4 μm diam. *Cystidioles* present. *Dendrohyphidia* present in hymenium. *Basidia* clavate, with four large sterigmata and a basal clamp connection, 18–21 × 4.5–6 μm; basidioles in shape similar to basidia, but smaller. Some irregular-shaped crystals present among hymenium. *Basidiospores* oblong ellipsoid to cylindrical, hyaline, thin-walled, smooth, IKI–, CB–, (6–)6.4–7.9(–8.2) × (2.5–)2.9–3.5(–4) μm, L = 7.17 μm, W = 3.17 μm, Q = 2.23–2.26 (*n* = 60/2).

Specimens examined: China: *Guangdong:* Zhanjiang, Campus of Guangdong Ocean University, N 40° 00′, E 116° 21′, on living tree of palm, 4 June 2019, *Y.C. Dai 19693* (BJFC031369). *Hainan:* Haikou, Jinniuling Park, N 20° 01′, E 110° 32′, on dead palm, 7 November 2020, *Y.C. Dai 21948* (BJFC035847); Sanya, Fairyland, N 18° 18′, E 109° 12′, on dead palm, 15 November 2020, *Y.C. Dai 22099* (BJFC035991). Vietnam: Ho Chi Minh, Reunification Palace, N 10° 77′, E 106° 69′, on dead palm, 10 October 2017, *Y.C. Dai 18296* (BJFC025818).

*Pseudogrammothele* Y.C. Dai, W.L. Mao & Yuan Yuan, gen. nov.

MycoBank no.: MB 846836.

*Etymology: Pseudogrammothele* (Lat.): refers to the genus resembling *Grammothele*.

*Type: Pseudogrammothele separabillima* (H.S. Yuan) Y.C. Dai, W.L. Mao & Yuan Yuan.

*Description: Basidiome* annual, resupinate, easily separate from the substrate, leathery when fresh soft corky when dry. Pore surface yellowish brown to pale luteous. *Subiculum* duplex with a distinct black line separating the two layers. Hymenium restricted to the base of tubes. *Hyphal system* dimitic; generative hyphae with clamp connections; skeletal hyphae IKI–, CB + . *Basidiospores* oblong ellipsoid, hyaline, thin-walled, smooth, usually with a large guttule, IKI–, CB + . Causing a white rot, growing on fallen angiosperm twig.

**Fig. 13:**
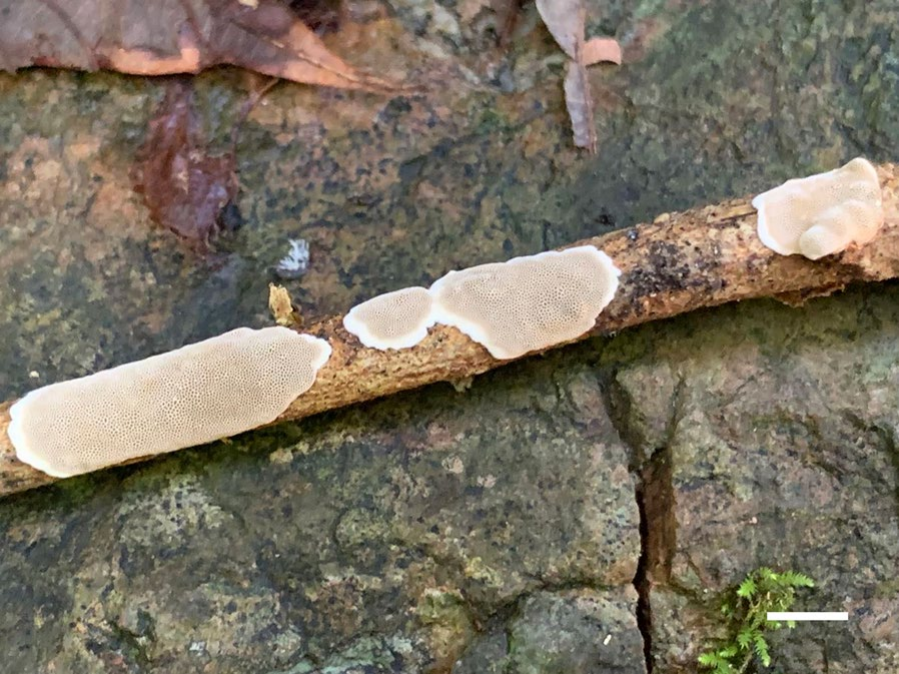
Basidiome of *Pseudogrammothele separabillima* (*Y.C. Dai 22599*). Bar = 1.0 cm

*Pseudogrammothele separabillima* (H.S. Yuan) Y.C. Dai, W.L. Mao & Yuan Yuan, comb. nov. (Fig. 13).

MycoBank no.: MB 846837.

*Basionym: Grammothele separabillima* H.S. Yuan, *Phytotaxa* 213: 50 (2015).

*Description: Basidiome* annual, resupinate, resupinate, effused, often elongated along thin branches, easily separate from the substrate, leathery when fresh, soft corky when dry, Sterile margin white. Pore surface yellowish brown to pale luteous; pores distinct, entire, angular, 4–6 per mm; dissepiments thin, finely pruinose. *Subiculum* duplex, upper layer concolourous with pore surface, lower layer dark brown. Hymenium restricted to the base of tubes. *Hyphal system* dimitic; generative hyphae with clamp connections; skeletal hyphae IKI–, CB + . *Dendrohyphidia* present. Basidia clavate, bearing four sterigmata and a basal clamp connection, 23–31 × 8–12 μm; basidioles in shape similar to basidia, but slightly smaller. *Basidiospores* oblong ellipsoid, hyaline, thin-walled, smooth, usually with a large guttule, IKI–, CB + , 9.8–11.4 × 6.4–7.3 μm.

*Specimens examined:* **China:** *Yunnan:* Jinghong, Xishuangbanna Botanic Garden, N 22° 1′, E 100° 54′, on fallen angiosperm branch, 7 July 2021, *Y.C. Dai 22599* (BJFC037173); Mengla County, Yulinggu Forest Park, N 21° 27′, E 101° 34′, on fallen angiosperm twig, 4 July 2021, *Y.C. Dai 22568* (BJFC037142).

**Fig. 14:**
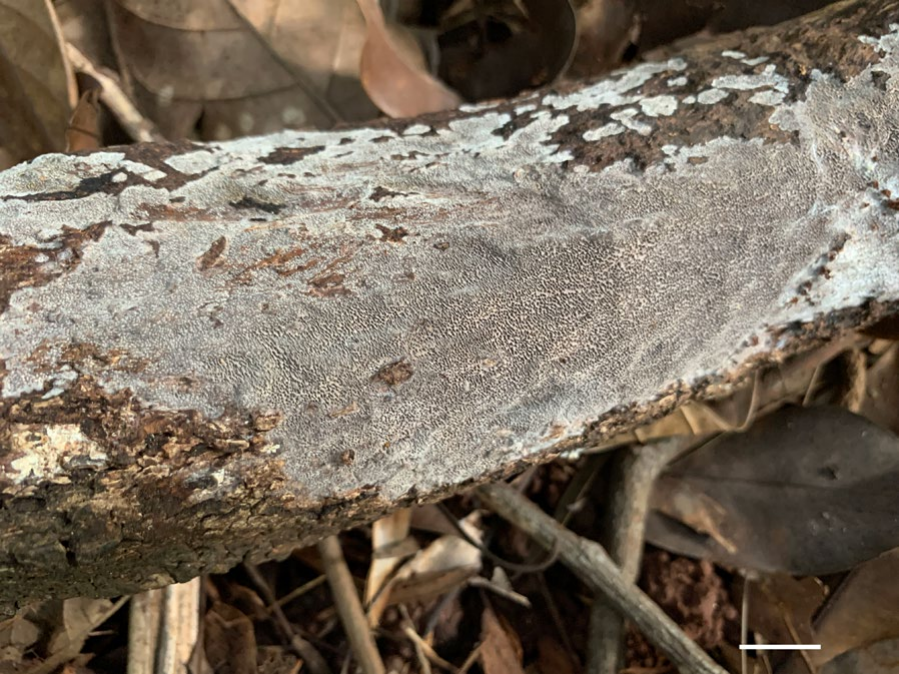
Basidiome of *Grammothele duportii* (*Y.C. Dai 21932*). Bar = 1.0 cm

**Fig. 15:**
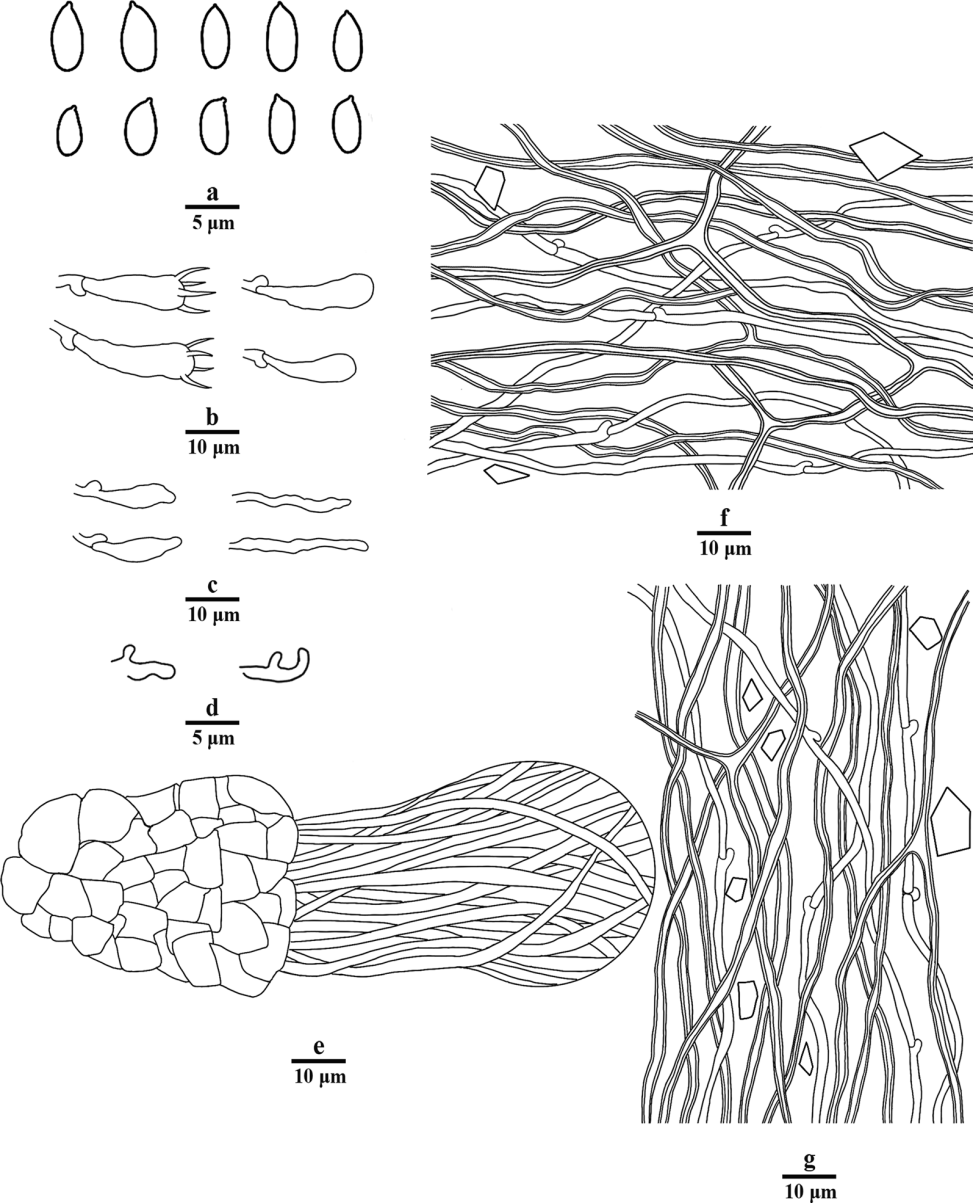
Microscopic structures of *Grammothele duportii* (*Y.C. Dai 21932*). a. Basidiospores; b. Basidia and basidioles; c. Cystidioles; d. Dendrohyphidia; e. A hyphal peg; f. Hyphae from subiculum; g. Hyphae from trama

*Grammothele duportii* (Pat.) Y.C. Dai, W.L. Mao & Yuan Yuan, comb. nov. (Figs. 14 and 15).

MycoBank no.: MB 846838.

*Basionym: Porogramme duportii* Pat., *Bull. Soc. mycol. Fr.* 29: 208, 1913; as '*duporti*'.

*Description: Basidiome* annual resupinate, adnate, corky to coriaceous, without special odor or taste when fresh, becoming hard corky and light in weight upon drying, to 9.5 cm long, 2.5 cm wide, and 1.4 mm thick at center; sterile margin narrow to almost lacking. Pore surface grayish white to sordid gray or violet gray; pores angular to irregular, 2–3 per mm; dissepiments thin, entire to lacerate. *Hyphal pegs* frequent, dotted-looking. Hymenium present at both the vertical tube-walls and the base of tubes. *Subiculum* buff, corky, becoming dark and resinous with age, to 0.6 mm thick. *Tubes* short, rigid corky when dry, to 0.8 mm long, under a lens the walls almost black, the bottom of tubes with white mycelia. *Hyphal system* dimitic; generative hyphae with clamp connections; skeletal hyphae IKI–, CB–; tissues darkening in KOH. *Subicular* generative hyphae infrequent, hyaline, thin-walled, rarely branched, flexuous, 2–2.5 µm diam; skeletal hyphae dominant, pale to dark brown, thick-walled with a narrow to medium lumen, frequently branched, strongly flexuous, interwoven, 2–3 µm diam. *Tramal* generative infrequent, hyaline, thin-walled, moderately branched, 1.5–2 µm diam; skeletal hyphae dominant, pale to dark brown, thick-walled with a narrow lumen to subsolid, frequently branched, mostly flexuous, interwoven, 1.5–3 µm diam. *Dendrohyphidia* frequently present, hyaline, thin-walled, moderately to strongly branched. *Hyphal pegs* often apically encrusted with large crystals, some hyphal pegs projected from tube trama or submerged in trama. *Cystidia* absent, two kinds of cystidioles present, one fusoid, slightly smaller than the basidioles, another hyphal-like, occurring in the hymenium, simulating narrow and cylindrical cystidioles. *Basidia* clavate, with four sterigmata and a basal clamp connection, 18–24 × 4–7 µm; basidioles dominant, in shape similar to basidia, but smaller. Big rhomboid or polyhedric crystals frequently present among hymenium. *Basidiospores* oblong ellipsoid tapering at apiculus, hyaline, thin-walled, smooth, IKI–, CB–, (5.2–)5.3–7(–7.2) × (2–)2.1–2.9(–3) µm, L = 6.03 µm, W = 2.48 µm, Q = 2.43–2.45(*n* = 60/2).

*Specimens examined:* **China:** *Hainan:* Changjiang County, Bawangling Nature Reserve, N 19° 05′, E 109° 05′, on fallen angiosperm trunk, 10 May 2009, *B.K. Cui 6539* (BJFC004392); Haikou, Jinniuling Park, N 20° 01′, E 110° 32′, on fallen angiosperm branch, 7 November 2020, *Y.C. Dai 21932* (BJFC035831). Singapore: Bukit Timah Nature Reserve, N 1° 35′, E 103° 77′, on fallen angiosperm trunk, 19 July 2017, *Y.C. Dai 17821* (BJFC025353); on rotten wood, 20 July 2017, *Y.C. Dai 17878* (BJFC025410).

## Discussion

Our phylogeny confirmed the close relationship among *Porogramme, Grammothele, Epithele, Theleporus*, and *Tinctoporellus* (Fig. 1), and six clades were formed. The clade A is named as *Porogramme* clade, and the type species of *Porogramme* (*P. albocincta*) and *Tinctoporellus* (*T. epimiltinus*) nested in the clade. *Porogramme* (1900) is an earlier name than *Tinctoporellus* (1979) and has a priority, so, we merge *Tinctoporellus* into *Porogramme.* The clade B is named as *Cyanoporus* clade. Species growing on monocotyledons (palm, bamboo etc.) are included in this clade. Although *Cyanoporus fuligo* was treated as *Grammothele fuligo* and *Porogramme fuligo*, but our phylogeny shows *Cyanoporus* forms an independent clade closely related to *Porogramme*, *Grammothele*, *Epithele*, and *Theleporus.* So, the genus *Cyanoporus* is proposed to accommodate *Cyanoporus fuligo* and related taxa. The clade C is named as *Grammothele* clade, and its type species, *G. lineata*, is included in the clade. The clade D is named as *Epithele* clade, most species with DNA data in *Epithele* are included in the clade, including the type species *Epithele typhae.* The clade E is named as *Theleporus* clade, most species in *Theleporus* are included in the clade although DNA data of its type species, *T. cretaceus*, are unavailable. The clade F is named as *Pseudogrammothele* clade, the single species *Pseudogrammothele separabillima* was originally described in *Grammothele* due to its anatomical characteristics fit the definition of the genus (resupinate basidiome with brownish pore surface, a dimitic hyphal system with hyaline to yellowish skeletal hyphae and the presence of dendrohyphidia, Yuan 2015). However, easily separating from the substrate, the distinct pores, duplex subiculum and large and cyanophilous basidiospores with guttules indicate the difference from *Epithele, Grammothele*, *Porogramme*, and *Theleporus* (Nakasone 2013; Yuan 2015)*.* Moreover, the divergence time estimation shows *P. separabillima* diverged at 79.1 Mya and evolved from the same ancester with *Polyporus squamosus*. Thus, the genus *Pseudogrammothele*, is proposed to accommodate *Pseudogrammothele separabillima.*

Recent molecular phylogenies demonstrated that hymenophore is not a key feature for separation of genera, and species with corticioid and poroid hymenophore are nested in the same genus, for instance, *Antrodia* (Runnel et al. 2019) and *Hymenochaete* (He and Dai 2012). Our phylogeny on *Porogramme, Tinctoporellus, Grammothele*, and *Theleporus* reveals the similar conclusion, the corticioid genus *Porogramme* and poroid genus *Tinctoporellus* nested in the same clade.

Phylogenetically, *Porogramme austroasiana* formed an independent lineage (Fig. 1). Morphologically, *Porogramme austroasiana* resembles *Grammothele lacticolor* Ryvarden by sharing resupinate basidiome with poroid hymenophore, approximately the same-sized pores and basidiospores (2–5 per mm, 3.8–4.5 × 2.1–2.5 µm vs. 3–4 per mm, 3–4 × 2–2.5 µm). However, *G. lacticolor* differs from *P. austroasiana* by the presence of hyphal pegs, reddening substrate, weakly dextrinoid skeletal hyphae, and distributed in central America (Ryvarden 2015).

Morphologically, *Porogramme cylindrica* may be confused with *P. austroasiana* and *P. bubalina* in having approximately the same pores size (2–5 per mm). However, the latter two species have distinctly smaller basidiospores (3.8–4.5 × 2.1–2.5 µm in *P. austroasiana*, 4.7–5.4 × 2.8–3.3 µm in *P. bubalina* vs. 8–10 × 3.1–3.8 µm in *P. cylindrica,* Yuan and Wan 2012). *Porogramme yunnanensis* is similar to *P. hinnulea* in having the same-sized pores (4–6 per mm), the presence of dendrohyphidia and ellipsoid basidiospores, but the latter has a dimitic hyphal system and wider basidiospores (4.5–5.2 × 2.5–3 µm vs. 3.7–5.0 × 2.0–2.6 µm, Yuan and Wan 2012).

Phylogenetically, *Porogramme cylindrica* is closely related to *P. yunnanensis* (Fig. 1), but the former has a dimitic hyphal structure and the absence of cystidioles, while the latter has a monomitic hyphal system and the presence of fusoid cystidioles.

Phylogenetically, *Porogramme subargentea* formed an independent lineage (Fig. 1) with a robust support (100% ML and 1.00 BPP). Morphologically, *P. subargentea* share similar cylindric basidiospores with *P. cylindrica*, but the latter species has non-dextrinoid skeletal hyphae and longer basidiospores (8–10 × 3.1–3.8 vs. 5.2–8.3 × 2.6–3.1 µm, Rajchenberg 1983).

In our phylogeny, a sample of *Porogramme albocincta* (FP102875sp) from Puerto Rico formed an independent lineage with *Porogramme micropora* (WX2014-116) from Brazil with a relatively high support (88% ML). Although the voucher specimen of *Porogramme albocincta* (FP102875sp) was not examined morphologically, *P. micropora* may be confused with *P. albocincta* due to the dark bluish gray basidiome and very small pores (8–20 per mm) according to Ryvarden (1979) and Hyde et al. (2019), we treated *P. albocincta* (FP102875sp) as ‘*P. micropora*’ based on our phylogeny.

*Cyanoporus fuligo* was originally described from Sri Lanka on dead palm, and Ryvarden has a general description of the species (Ryvarden 1979). According to our study it seems to be a species complex, and two species are existed, samples (Dai 21117, 21936, 21937, 21950) with small pores formed an independent lineage with a robust support (100% ML and 1.00 BPP), and samples with relatively big pores (Dai 19693, 21948, 22099, 22117) formed another independent lineage with a robust support (100% ML and 1.00 BPP). The pores in the former lineage are 8–12 per mm which are almost invisible to the naked eye, so, it fits the original description of *Polyporus fuligo*: “pores quite invisible to the naked eye, so that it looks like a *Corticium*”. Therefore, samples of Dai 21117, 21936, 21937 and 21950 are treated as *Cyanoporus fuligo.*

Six taxa were treated as synonyms of *Cyanoporus fuligo* (Ryvarden and Johansen 1980, MycoBank: https://www.mycobank.org/page/Simple%20names%20search; Index Fungorum: http://www.speciesfungorum.org/GSD/GSDspecies.asp?RecordID=314701). Among them, *Porogramme camptogramma* Pat. was described on bamboo from northern Vietnam (Patouillard 1913). Its original description as “pores about 6 per mm, spore oblong to oblong ellipsoid, 5.5–8 × 2.5–3.5 µm”. In our study, samples of Dai 18296, 19693, 21948, 22099, 22117 from tropical China and Vietnam have pores of 5–8 per mm and basidiospores of 6.4–7.9 × 2.9–3.5 µm. These important features fit *Porogramme camptogramma* well. So, we treated these samples as *Cyanoporus camptogramma.*

*Grammothele lineata* was originally described from Cuba (Berkeley and Curtis 1869), and Ryvarden and Johansen (1980) give a general description of the species. According to our study, it seems to be a species complex, and three species are existed and formed three independent lineages (Fig. 1): *G. lineata* sensu stricto from tropical America (samples Dai 18485 and WX2014-208 from Brazil, molecular data from its type locality are unavailable so far),* G. denticulata* Y.C. Dai & L.W. Zhou from China (Zhou and Dai 2012) and a taxon represented by Southeast Asian samples of Cui 6539, Dai 17821 and 21932.

Nine taxa were treated as synonyms of *Grammothele lineata* (Ryvarden and Johansen 1980, MycoBank: https://www.mycobank.org/page/Simple%20names%20search; Index Fungorum: http://www.speciesfungorum.org/GSD/GSDspecies.asp?RecordID=168936), among them, *Porogramme duportii* was described from northern Vietnam (Patouillard1913). Its original description as “pores chalk white with grayish reflection, about 0.25 mm thick, subhymenial white, pores diameter 200–250 µm (dissepiments not included), hyphal pegs present in tube walls”. Our Asian samples of Cui 6539, Dai 17821 and 21932 fit *P. duportii* well, so, we combined this taxon as *Grammothele duportii*. *G. duportii* are very similar to *G. lineata*, and the Chinese samples were treated as the latter previously (Dai et al. 2011), but differs from *G. lineata* by the slightly wider basidiospores (5.3–7 × 2.1–2.9 µm vs. 4.5–6 × 1.5–2.5 µm, Ryvarden and Johansen 1980) and the presence of two kinds of cystidioles.

In addition, three new combinations *Porogramme brasiliensis*, *P. micropora*, and *P. venezuelica* are proposed based on phylogenetic analysis only, because we did not study their voucher materials (Fig. 1). *Porogramme aurantiaca*, another new combination from tropical America (samples Dai 17401 and WX2014-115), has reddish zones beneath basidiome which matches the morphological characteristics of *Porogramme*.

According to our phylogeny, the genus *Epithele* formed an independent clade with a robust support (100% ML and 1.00 BPP), and another related genus *Theleporus*, a few known species in the genus with molecular sequences formed an independent clade related to *Porogramme, Grammothele*, and *Epithele* with a relatively low support (51% ML). However, its type species, *Theleporus cretaceus* from South Africa (Fries 1849), is not analyzed because its DNA data are not available so far. To confirm its affinity, more samples, especially material of *T. cretaceus* from type locality is badly needed. Moreover, the two genera,*Theleporus*, and *Epithele*, occurred in a mean stem age of 79.2 Mya and 72.9 Mya, respectively, the estimation results are in accord with the results carried out by Ji et al. (2022) that the mean stem ages of the six major clades of Polyporus are approximately 47–60 Mya (Fig. 2).

Recently, dating analyses have provided a deep insight into the evolution of *Polyporales* (Song and Cui 2017; Zhao et al. 2017; Ji et al. 2022). Our analysis of divergence time estimation suggests that *Polyporales* occurred in a mean stem age of 187.6 Mya and *Polyporaceae* of which species mostly growing on the angiosperm woods possibly emerged in a mean stem age of 152.9 Mya (Fig. 2). Moreover, species preferring to grow on gymnosperm woods in *Polyporales* emerged in an earlier mean stem age of 164.6 Mya. Considering many botanists confirmed the crown age for the angiosperms was at least 160 Mya, our divergence time estimation of the six clades of *Porogramme* and related genera corresponded with the previous study (Ji et al. 2022) that the mean stem ages of six major clades of *Polyporus* are approximately 47–60 Mya, the mean stem ages of the six genera we recognize all earlier than 50 Mya (Fig. 2), thus, it is reasonable to recognize the six clades (*Porogramme*, *Grammothele*, *Cyanoporus*, *Epithele*, *Theleporus*, and *Pseudogrammothele*) as independent genera.

## Conclusion

Six clades represent *Porogramme*, *Grammothele*, *Cyanoporus*, *Epithele*, *Theleporus*, and *Pseudogrammothele* are recognized based on phylogenetic analysis and morphological examination on samples from tropical or subtropical Asia and America (Fig. 1), among them *Cyanoporus* and *Pseudogrammothele* are proposed as new genera. *Tinctoporellus* is merged into *Porogramme* because the type species of both genera are nested in the same clade. Three new species of *Porogramme* are described and illustrated, and the definition of the genus is revised. Twelve new combinations in *Cyanoporus*, *Grammothele*, *Porogramme*, and *Pseudogrammothele* are proposed. The molecular clock analyses also support the six clades as independent genera due to the mean stem ages of the six genera we recognize all earlier than 50 Mya (Fig. 2).

Based on morphological and phylogenetic analyses on *Porogramme*, *Grammothele*, *Cyanoporus*, *Epithele*, *Theleporus*, and *Pseudogrammothele*, their phylogenetic relationships and general morphological characteristics are outlined. In our phylogeny, the six genera belong to *Polyporales* and being closely related with each other (Fig. 1). Among them, *Cyanoporus*, proposed as a new genus, is characterized by its bluish pores, hymenium restricted to the base of tubes, rarely branched and subparallel skeletal hyphae along tubes and usually growing on monocotyledons. Another new genus, *Pseudogrammothele*, characterized by its basidiome is easily separated from the substrate, distinct pores, duplex subiculum and large and cyanophilous basidiospores with guttules, so, differs it from *Epithele, Grammothele*, *Porogramme*, and *Theleporus. Epithele* and *Theleporus* are similar by sharing hymenia restricted to the base of tubes, while *Epithele* is distinguished by its basically smooth hymenophore, hyphal pegs composed of trama hyphae and usually thick-walled basidiospores. Hyphal pegs are also common in most species of *Grammothele*, while *Grammothele* has more or less poroid hymenophore and thin-walled basidiospores. Upon the recombination of *Tinctoporellus*, *Porogramme* and several species traditionally belong to *Grammothele*, *Porogramme* is characterized by hymenium present at both the vertical tube-walls and the base of tubes or restricted to the base of tubes, a monomitic or dimitic hyphal system, generative hyphae with clamp connections or with both clamp connections and simple septa, hyphae dextrinoid or not and reddening substrate in most species.

## Data Availability

All sequence data generated for this study can be accessed via GenBank: https://www.ncbi.nlm.nih.gov/genbank/. Alignments are available at TreeBase (http://www.treebase.org; submission ID: 29977).

## Abbreviations

BI: Bayesian inference
BJFC: Herbaria of the Institute of Microbiology, Beijing Forestry University
BPP: Bayesian posterior probability
BS: Bootstrap
CB: Cotton blue
GTR + I + G: General time reversible + proportion invariant + gamma
IKI: Melzer’s reagent
ITS: Nuclear ribosomal internal transcribed spacer
KOH: 5% Potassium hydroxide
MCC: Maximum clade credibility
ML: Maximum likelihood
Mya: Million years ago
PCR: Polymerase chain reaction
RPB1: Largest subunit of RNA polymerase II
RPB2: Second largest subunit of RNA polymerase II
TEF1: Translation elongation factor 1-α
